# Variable-angle locking plate with or without double-tiered subchondral support procedure in the treatment of intra-articular distal radius fracture

**DOI:** 10.1007/s10195-014-0292-0

**Published:** 2014-06-19

**Authors:** Keikichi Kawasaki, Tetsuya Nemoto, Katsunori Inagaki, Kazunari Tomita, Yukio Ueno

**Affiliations:** Department of Orthopaedic Surgery, Showa University School of Medicine, 1-5-8 Hatanodai, Shinagawa-ku, Tokyo, 142-8666 Japan

**Keywords:** Distal radius fracture, Locking plate, Double-tiered subchondral support

## Abstract

**Background:**

Double-tiered subchondral support (DSS) procedure is two-row fixation in which proximal screws support the dorsal subchondral bone, whereas distal screws support the volar central subchondral bone, using the volar variable-angle locking plate to achieve better anatomical reduction. We examined whether DSS improves clinical outcome, complication rate, and loss of correction for dorsally displaced Arbeitsgemeinschaft für Osteosynthesefragen (AO) type C3 distal radius fractures.

**Materials and methods:**

We reviewed dorsally displaced intra-articular AO C3-type distal radius fractures treated at our institutions with a variable-angle volar locking plate. We assessed 49 patients (27 DSS; 22 non-DSS) treated with volar locking plates, with a mean age of 59.9 years and average follow-up of 20.2 months (range 12–56 months). We evaluated differences in functional outcome, complication rates, and loss of correction between groups using radiographic parameters.

**Result:**

There were no differences in clinical outcome and complications. Final volar tilt and ulnar variance were better maintained in the DSS group (*P* = 0.01 and 0.03). Change in volar tilt of the non-DSS group was more than that of the DSS group (*P* = 0.00).

**Conclusion:**

Though there were no significant differences in clinical outcomes, we identified a significant reduction in final volar tilt, ulnar variance, and change in volar tilt. DSS procedure is useful to avoid correction loss when treating unstable C3 distal radius fractures and thus would reduce posttraumatic arthrosis.

**Level of evidence:**

Level IV.

## Introduction

**Fig. 1:**
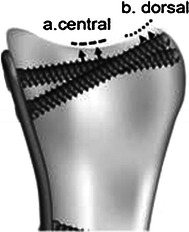
Lateral view of distal radius with a variable-angle volar plate using the double-tiered subchondral support procedure. Proximal screws support the dorsal subchondral bone; distal screws support the central subchondral bone

In intra-articular distal radius fractures, posttraumatic arthrosis is directly related to the degree of anatomical reduction [1, 2]. AO type C3 distal radius fractures are the most difficult to reduce and stabilize in the anatomical position because of the multifragmentary nature, high incidence of correction loss, and poorer prognosis for these patients [3]. The double-tiered subchondral support (DSS) procedure has been reported to keep fractures of the distal radius in anatomical position when using the volar locking plate system [4]. With DSS, proximal screws support the dorsal subchondral bone from the proximal screws, whereas distal screws support the central subchondral bone from the distal row (as determined from a lateral view; Fig. 1). However, there is no evidence to support the usefulness of the DSS procedure for treating unstable distal radius fractures.

In this study, we examined the hypothesis that the DDS procedure in variable-angle volar locking plates could improve clinical outcome, complications, and loss of correction in the short term for patients with dorsally displaced Arbeitsgemeinschaft für Osteosynthesefragen (AO) type C3 intra-articular distal radius fractures.

## Materials and methods

We reviewed all cases of AO type C3 distal radius fractures treated at our institutions with volar locking plate from 2008 to 2015 with a follow-up time >12 months. Patients with a period from injury to operation >14 days were excluded. In addition, one patient who presented with an ipsilateral carpal bone fracture was excluded. The 49 patients consisted of 11 men and 38 women, with a mean age of 59.9 years (range 23–85). The group treated with DSS comprised 27 patients (four men, 23 women), and the group treated without DSS comprised 22 patients (seven men, 15 women). Patients characteristics are summarized in Table 1. There were no significant differences between groups except for age, with DSS patients being significantly older.

**Table 1:** Patient characteristics

	DSS group (*n* = 27)	Non-DSS group (*n* = 22)
Age* (*P* = 0.02)	64.3 (23–85)	54.5 (26–83)
Gender (male:female)	4:23	7:15
Dominant hand injured	12	8
Follow-up period	20.2 (12–53)	20.1 (12–56)
Ulnar styloid fracture	19	15

* Significant difference between groups (*P* = 0.02)

**Fig. 2:**
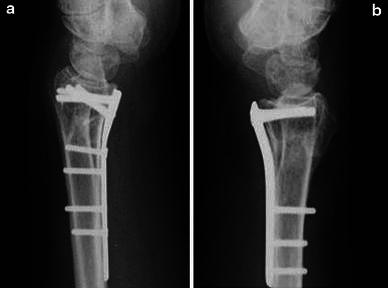
Postoperative lateral X-ray in the double-tiered subchondral support (DSS) (**a**) and non-DSS (**b**) groups

Patients were treated with open reduction and internal fixation (ORIF) with volar locking plates, with or without DSS. For all patients, we used the APTUS radius 2.5 fixation system (Medartis, Basel, Switzerland) with variable-angle volar locking plate, with a thickness of 1.6 mm and ±15° range of variable locking angles. In the DSS procedure, proximal screws were inserted toward the dorsal subchondral bone, and distal screws were inserted toward the central subchondral bone (Fig. 1). To be included in the DSS group, patients needed to be treated with more than two proximal and distal screws (Fig. 2a). The remaining patients formed the non-DSS group (Fig. 2b). Radiographic parameters, including volar tilt (VT), radial inclination (RI), and ulnar variance (UV) were assessed on the operative day and at final follow-up. We evaluated range of motion, percent grip power, and Mayo wrist score [5] to determine clinical outcome.

During postoperative care, wrists were immobilized in a volar splint for 1 week, and active and passive finger-motion exercises were started on the second postoperative day. Active motion of the wrist was undertaken from 1 week postoperatively. Passive movements were permitted after bony union was confirmed by surgeons. We evaluated functional, clinical, and radiographic outcomes and compared findings between groups. Activities of daily life (ADL) were assessed using the Mayo wrist scoring system by surgeons. We performed statistical analysis using Fisher’s exact test for categorical data and the independent *t* test for continuous data.

## Results

Results are summarized in Table 2, and there were no significant differences between groups.

**Table 2:** Clinical outcomes measures

	DSS group (*n* = 27)	Non-DSS group (*n* = 22)	*P* value
Range of motion			
Flexion	68.0 ± 12.0	65.5 ± 12.3	0.48
Extension	74.8 ± 10.4	74.8 ± 11.6	0.99
Pronation	87.0 ± 5.9	86.4 ± 7.1	0.72
Supination	89.6 ± 1.3	88.2 ± 5.7	0.25
Percent grip power	97.3 ± 24.1	88.3 ± 20.3	0.17
Cooney score	91.1 ± 5.9	91.4 ± 8.8	0.91

Data are mean ± standard deviation. There were no significant differences between groups

Radiographic parameters are summarized in Table 3. Postoperative UV in the DSS group was smaller and final VT better maintained in the DSS group; change in VT was greater in the non-DSS group.

**Table 3:** Radiological outcomes measures

	DSS group (*n* = 27)	Non-DSS group (*n* = 27)	*P* value
Postoperative volar tilt (°)	7.5 ± 4.9	6.2 ± 4.8	0.35
Postoperative radial inclination (°)	24.0 ± 3.9	22.7 ± 3.1	0.21
Postoperative ulnar variance (mm)	0.2 ± 1.2	−0.7 ± 1.6	0.03*
Final volar tilt (°)	8.0 ± 5.8	3.8 ± 5.0	0.01*
Final radial inclination (°)	24.8 ± 4.2	23.0 ± 3.3	0.09
Final ulnar variance (°)	1.0 ± 1.1	0.2 ± 1.6	0.03*
Change in volar tilt (°)	+0.5 ± 1.9	−2.4 ± 3.4	0.00*
Change in radial inclination (°)	+0.8 ± 2.4	+0.2 ± 2.4	0.41
Change in ulnar variance (mm)	+0.8 ± 0.9	+0.8 ± 1.2	0.94

Data are mean ± standard deviation

* Significant difference

Postoperative complications occurred in two patients: one in the DSS group had a mild subcutaneous infection, and one in the non-DSS group developed complex regional pain syndrome type 1. Both patients were treated conservatively. None of the patients had delayed union or nonunion.

## Discussion

AO type C3 distal radius fractures are the most unstable fractures, and some groups have indicated the limitation of treatment with volar locking plate alone, using additional K wires, and dorsal-plating fixation for patients with these types of fractures [6–9]. Another group reported that additional screws and K wires were not sufficient in these cases and that additional bone grafting should be performed for metaphyseal bone loss [10]. In our study, though there were no significant differences in clinical outcomes, we identified a significant reduction in final VT, RI, and change in VT. Distal screws support the central subchondral bone and transmit the axial force from the subchondral bone to the intact diaphyseal bone [11]. We believe that this transmission of force maintained anatomical reduction of the lunate facet, thus achieving good clinical outcomes. However, in non-DSS cases in which distal screws only were used, we observed a VT correction loss of after early mobilization in AO type C3 fractures compared with the DSS group. We hypothesize that in multifragmentary articular fractures, distal screws alone are not sufficient to catch the dorsal subchondral bone. As such, dorsal articular subchondral bone should be supported with proximal screws. In the DSS group, we believe that proximal screws transferred the dorsal articular load to the implant and the diaphyseal bone. Although correction loss of VT did not affect short-term clinical outcomes, it is likely to affect posttraumatic arthrosis; a longer follow-up period is required to determine if this is the case.

In this study, RI in the DSS group was better maintained than in the non-DSS group. Stanbury et al. showed the superiority of a variable-angle locking plate for capturing a distal radial styloid compared with a fixed-angle plate in biomechanical study [12]. A variable locking plate using the DSS procedure might capture a distal radial styloid fragment better than a non-DSS procedure. The overall complication rate in our study was 4.1 %, with complications occurring in each group and no significant difference observed. A similar complication ratio of 3 % was observed in another short-term study with a mean follow-up of 12 months [13]. Flexor tendon rupture after volar plate fixation has been reported by some authors [14, 15]. In some severe intra-articular fractures, it was necessary to place the plate distally from the watershed line [16], which is a transverse ridge that closes the concave surface of the volar radius distally. If the plate is placed above or distally from this line, it increases the risk of flexor tendon rupture. We believe that the risk of flexor pollicis longus (FPL) rupture would be decreased by using a thinner volar plate. In cadaveric models, insertion of screws in all available holes in the distal fragment of a variable-angle volar plate showed the highest biomechanical stability [17]. In our series, we routinely removed the plate system after bony union to take into consideration the risks to flexor tendons.

There were several limitations to this study. We could not compare operation time between groups because we simultaneously performed operations targeting accompanied disorders, such as ulnar styloid fracture. The DSS procedure required longer fluoroscopy time because of the careful attention required to position screws. Also, three of 27 patients in the DSS group and one in the non-DSS group underwent artificial bone grafting, which is useful for AO type C3 distal radius fractures for articular surface reconstruction [6]. Finally, this was a retrospective comparative study; a prospective randomized study is required to examine the effectiveness of the DSS procedure. A larger patient cohort and longer follow-up are necessary to evaluate the usefulness of the DSS procedure.

In conclusion, patients with AO type C3 distal radius fractures treated with the DSS procedure had a reduced change in VT than patients treated with the non-DSS method. We believe that the DSS procedure improves loss of VT correction and would help prevent posttraumatic arthrosis in the long term.
