# Erratum to: Variable-angle locking plate with or without double-tiered subchondral support procedure in the treatment of intra-articular distal radius fracture

**DOI:** 10.1007/s10195-014-0313-z

**Published:** 2014-07-31

**Authors:** Keikichi Kawasaki, Tetsuya Nemoto, Katsunori Inagaki, Kazunari Tomita, Yukio Ueno

**Affiliations:** Department of Orthopaedic Surgery, Showa University School of Medicine, 1-5-8 Hatanodai, Shinagawa-ku, Tokyo, 142-8666 Japan

## Erratum to: J Orthopaed Traumatol DOI 10.1007/s10195-014-0292-0

The author would like to correct the following error in the publication of the original article:

In the discussion section, eighth sentence of the first paragraph should read as:

“In our study, though there were no significant differences in clinical outcomes, we identified a significant reduction in final VT, UV, and change in VT”.

